# Interleukin-23p19 Inhibitors in Inflammatory Bowel Disease: From Current Insights to Future Directions

**DOI:** 10.3390/jpm16020119

**Published:** 2026-02-14

**Authors:** Ilse A. Pool, Antonius T. Otten, Jos G. W. Kosterink, Gerard Dijkstra, Paola Mian, Arno R. Bourgonje

**Affiliations:** 1 Department of Gastroenterology and Hepatology, University Medical Center Groningen, University of Groningen, Hanzeplein 1, 9713 GZ Groningen, The Netherlands; i.a.pool@umcg.nl (I.A.P.); a.t.otten@umcg.nl (A.T.O.); gerard.dijkstra@umcg.nl (G.D.); 2 Department of Clinical Pharmacy and Pharmacology, University Medical Center Groningen, University of Groningen, Hanzeplein 1, 9713 GZ Groningen, The Netherlands; j.g.w.kosterink@umcg.nl (J.G.W.K.); p.mian@umcg.nl (P.M.); 3 PharmacoTherapy, Epidemiology & Economics, Groningen Research Institute of Pharmacy, University of Groningen, Hanzeplein 1, 9713 GZ Groningen, The Netherlands; 4 Pharmacometrics Expertise Center of the Northern Netherlands, University Medical Center Groningen, University of Groningen, Hanzeplein 1, 9713 GZ Groningen, The Netherlands; 5 Department of Pediatrics, Beatrix Children’s Hospital, University Medical Center Groningen, University of Groningen, Hanzeplein 1, 9713 GZ Groningen, The Netherlands; 6 Department of Pharmaceutical, Technology and Biopharmacy, Groningen Research Institute of Pharmacy, University of Groningen, Hanzeplein 1, 9713 GZ Groningen, The Netherlands; 7 The Dr. Henry D. Janowitz Division of Gastroenterology, Department of Medicine, Icahn School of Medicine at Mount Sinai, 1468 Madison Avenue, New York, 10029 NY, USA

**Keywords:** inflammatory bowel disease, IL-23p19 inhibitors, biomarkers, precision medicine

## Abstract

Interleukin-23 (IL-23) is a pivotal cytokine driving intestinal inflammation in inflammatory bowel disease (IBD). The development of monoclonal antibodies selectively targeting the p19 subunit of IL-23, including risankizumab, mirikizumab and guselkumab, has significantly expanded the therapeutic landscape of IBD. Landmark phase 3 trials in Crohn’s disease (CD) and ulcerative colitis (UC) have demonstrated high efficacy and durable responses, followed by recent regulatory approvals across both indications. Notably, the SEQUENCE trial established the superiority of risankizumab over ustekinumab in achieving endoscopic and clinical endpoints in CD, underscoring the therapeutic value of IL-23p19 blockade and its differentiation from prior p40 inhibition. With additional agents in advanced development, IL-23p19 inhibitors are now emerging as a bona fide treatment class in IBD. Furthermore, IL-23p19 inhibitors display favorable safety profiles and convenient subcutaneous administration regimens, which broaden their applicability across diverse patient populations. However, key knowledge gaps remain regarding optimal treatment positioning, comparative effectiveness, and long-term disease outcomes. Precision medicine approaches will be crucial to fully exploit the potential of this drug class. For instance, early biomarkers can help monitor response, while future integration of serological and multi-omics biomarkers may enable the prediction of treatment success and guide personalized selection. This review summarizes the current knowledge base regarding IL-23p19 inhibitors in IBD, highlights their class effects and unique clinical value, and outlines a research agenda towards biomarker-driven and precision-guided use. Ultimately, IL-23p19-inhibition exemplifies how targeted immunotherapy and precision medicine can converge in order to reshape IBD management.

## 1. The Role of IL-23 in the Pathogenesis of IBD

**Figure 1:**
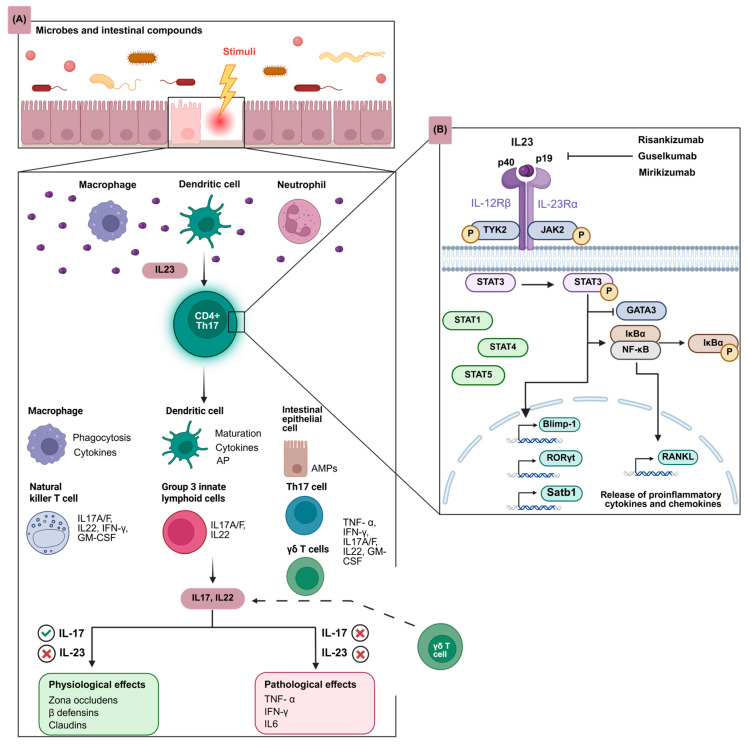
IL-23 production and pathway signaling in the intestinal mucosa. (**A**) Microbial and nonmicrobial compounds trigger IL-23 production by macrophages and DCs, and to a lesser extent by neutrophils. This promotes CD4^+^ Th17 cell proliferation. Activated cells release cytokines, including IL-17 and IL-22, which can also be produced independent of IL-23 signaling by γδ T cells and innate lymphoid cells. They exert both physiological effects and pathophysiological effects. (**B**) IL-23 (p19 and p40) binding to its receptor induces the phosphorylation of TYK2 and JAK2. This phosphorylates STAT3 and to a lesser extent STAT1, STAT4, and STAT5. Activated STAT3 induces the expression of RORγt, BLIMP-1 and Satb1 in the nucleus. STAT3 inhibits GATA3 and phosphorylates IκBα in the cytoplasm. NF-κB then translocates to the nucleus and induces the transcription of RANKL. Altogether, as a result, different proinflammatory cytokines and chemokines will be released. Abbreviations: BLIMP-1, B lymphocyte–induced maturation protein 1; CD4^+^ Th17 cells, CD4^+^ T helper 17 cells; DCs, dendritic cells; GATA3, GATA-binding protein 3; GM-CSF, granulocyte–macrophage colony-stimulating factor; IκBα, inhibitor of nuclear factor-κB alpha; IFN-γ, inter-feron γ; IL, interleukin; JAK2, Janus kinase 2; NF-κB, nuclear factor κ-light-chain-enhancer of ac-tivated B cells; RANKL, receptor activator of nuclear factor-κB ligand; RORγt, RAR-related orphan receptor γt; Satb1, special AT-rich sequence-binding protein 1; STAT, signal transducer and acti-vator of transcription; TNF-α, tumor-necrosis factor α; TYK2, tyrosine kinase 2.

Inflammatory bowel disease (IBD), including Crohn’s disease (CD) and ulcerative colitis (UC), is characterized by chronic ulcerative inflammation of the gastrointestinal tract. Although not completely understood, IBD pathophysiology entails a complex interplay between genetic predisposition, the host immune system, the gut microbiota and environmental factors including lifestyle and diet [1]. The therapeutic landscape of IBD has expanded significantly over the past decades with the introduction of biologics and small-molecule therapies [2]. Although these drugs have demonstrated efficacy and safety, primary and secondary loss of response occur frequently. Notably, primary loss of response rates range from 40 to 50%, while sustained remission at 12 months is achieved in approximately 30–50% of patients [3,4,5]. These numbers highlight the limitations of current biologic therapies and illustrate the need for the development of new biologics. A newly emerging and promising class of biologics is interleukin (IL)-23p19 inhibitors, including risankizumab, guselkumab, and mirikizumab. These agents have shown favorable efficacy and safety profiles in phase 3 clinical trials [6,7,8,9,10,11,12,13,14]. IL-23 is a heterodimeric cytokine within the IL-12 family and consists of two subunits, p19 and p40 [15]. IL-23 plays a pivotal role in the disease pathogenesis of IBD (Figure 1) [15]. Within the IL-23-dependent pathway, IL-23 is predominantly produced by macrophages and dendritic cells (DCs) in response to predominantly intestinal microbes. Subsequently, IL-23 binds to T-helper (Th) 17 cells and signal transduction is started, which finally results in the autophosphorylation of Janus kinase 2 (JAK2) and tyrosine kinase 2 (TYK2) [16]. The phosphorylated JAK2 will activate signal transducers and activators of transcription (STAT) 3 and, to a lesser extent, STAT 1, 4 and 5 [17]. Activated STAT3 translocates to the nucleus and can induce the expression of RAR-related orphan receptor γ t (RORγt), B lymphocyte-induced maturation protein 1 (BLIMP-1) and special AT-rich sequence-binding protein 1 (Satb1) [16]. In the cytoplasm, activated STAT3 will inhibit GATA-binding protein 3 (GATA3), thereby inhibiting regulatory T-cell function [16]. Moreover, the inhibitor of nuclear factor-κB alpha (IκBα) is phosphorylated and subsequently degraded by the proteasome [16]. In response, nuclear factor κ-light-chain-enhancer of activated B cells (NF-κB) translocates to the nucleus and induces the gene expression of the receptor activator of nuclear factor-κB ligand (RANKL) [16,17]. The expression of the aforementioned genes activates Th17 cells, as well as group 3 innate lymphoid cells, natural killer T cells, γδ T cells, intestinal epithelial cells, macrophages and DCs [18]. As a response, many different effector cytokines and chemokines will be secreted, including IL-17 and IL-22. These cytokines may have physiological effects, such as producing a protective barrier function by stimulating the production of zona occludens and claudins, and potential pathophysiological effects, such as enhancing further inflammatory responses through the release of tumor necrosis factor (TNF)-α, interferon (IFN)-γ and IL-6. As this inflammatory cascade is initiated by the secretion of IL-23, this pathway is called the IL-23-dependent pathway. Unlike the IL-23-dependent pathway, IL-17 and IL-22 can be produced independently of IL-23 by γδ T cells and innate lymphoid cells, thereby contributing to a protective barrier function. In this way, selective inhibition of IL-23p19 is hypothesized to preserve the protective functions of IL-23, while inhibiting its pathologic effects [18]. Specifically, a systematic review on IL-17 inhibitors, including secukinumab, ixekizumab and brodalumab, demonstrated that secukinumab and ixekizumab are associated with new-onset IBD and disease exacerbation in patients with CD or UC [19]. In contrast, brodalimumab did not trigger any safety signals.

## 2. Current Evidence for IL-23p19 Inhibitors

**Figure 2:**
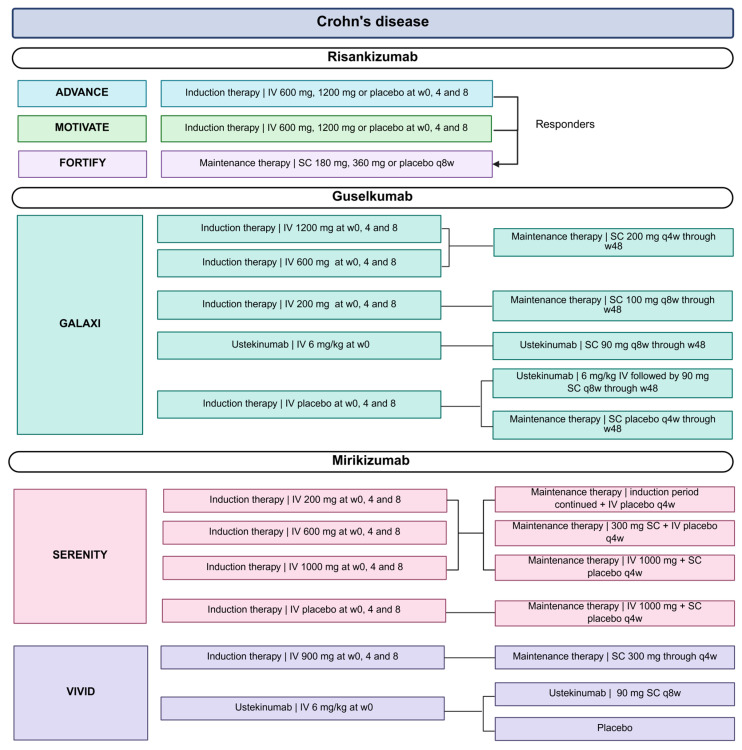
Overview of treatment arms and dosing regimens in clinical trials evaluating risankizumab, guselkumab and mirikizumab in CD. The ADVANCE, MOTIVATE and FORTIFY trials investigated risankizumab; GALAXI studied guselkumab; and SERENITY and VIVID evaluated mirikizumab. Abbreviations: CD, Crohn’s disease; IV, intravenous; kg, kilogram; mg, milligram; q, every; SC, subcutaneous; w, weeks.

**Figure 3:**
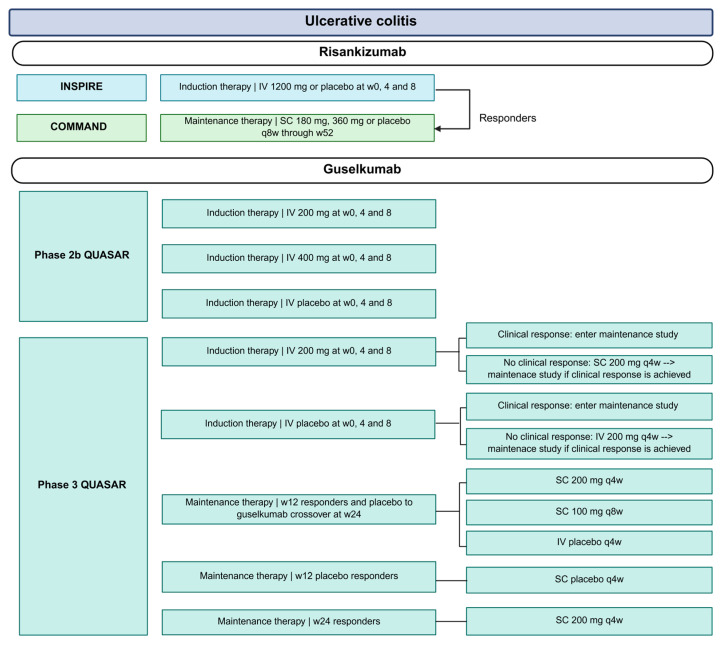
Overview of treatment arms and dosing regimens in clinical trials evaluating risankizumab, guselkumab and mirikizumab in UC. The INSPIRE and COMMAND trials investigated risankizumab; QUASAR studied guselkumab; and LUCENT evaluated mirikizumab. Abbreviations: IV, intravenous; mg, milligram; q, every; SC, subcutaneous; w, weeks.

Large phase 3 trials in CD and UC have firmly established the IL-23p19 inhibitors risankizumab, guselkumab and mirikizumab as effective, safe and tolerable therapies in moderate-to-severe IBD. An overview of the landmark trials and corresponding dosing regimens is provided in Figure 2 and Figure 3. In addition, Table A1 summarizes the primary and endoscopic and/or histologic outcomes of these key trials. Our Table A1 further extends Table A1 of a previously published review in *Gastroenterology* [18].

### 2.1. Clinical Trial Data for Classical Administration Routes of IL-23p19 Inhibitors

#### 2.1.1. Risankizumab

Risankizumab was approved by the Food and Drug Administration (FDA) and European Medicines Agency (EMA) for CD in 2022 and for UC in 2024, making it the first IL-23p19 inhibitor approved for both types of IBD. One early phase 2 trial studied the use of risankizumab in patients with CD. They found that risankizumab was more effective than placebo in inducing remission in patients with active CD [20]. The ADVANCE and MOTIVATE induction trials and the FORTIFY maintenance trial evaluated risankizumab in patients with moderate-to-severe CD [21,22]. All patients included in the MOTIVATE trial had experienced intolerance or inadequate response to at least one approved biologic for CD. The ADVANCE trial included patients with previous biologic therapy failure as well as those with inadequate response to conventional therapies. In the ADVANCE and MOTIVATE trials, risankizumab treatment led to higher rates of clinical remission combined with endoscopic response at week 12 compared with placebo. Clinical remission rates based on a Crohn’s Disease Activity Index (CDAI) were consistent. In MOTIVATE, response rates of 42.5% (600 mg), 40.3% (1200 mg) and 19.8% (placebo) were observed. ADVANCE showed response rates of 45.2% (600 mg), 41.6% (1200 mg) and 25.2% (placebo) (all, *p* < 0.001; Table A1). In MOTIVATE, endoscopic response rates of 28.8% (600 mg), 34.2% (1200 mg) and 11.2% (placebo) were observed. ADVANCE showed response rates of 40.3% (600 mg), 32.2% (1200 mg) and 12.0% (placebo) (all, *p* < 0.001; Table A1). Notably, no dose–response relationship was observed, as the 1200 mg dose did not show better response rates compared to the 600 mg dose. Patients in MOTIVATE and ADVANCE were re-randomized into the FORTIFY maintenance trial to receive subcutaneous (SC) risankizumab (180 mg or 360 mg every eight weeks) or placebo [22]. Outcomes were achieved significantly more often in patients treated with risankizumab compared with placebo. CDAI-defined clinical remission occurred in 55% of patients receiving SC 180 mg and 52% receiving SC 360 mg, versus 41% in the placebo group. Endoscopic response was observed in 47% of patients in both risankizumab dose groups, compared with 22% with placebo. Both risankizumab regimens were superior to placebo for endoscopic remission (30–39% vs. 13%; *p* < 0.01) and deep remission (25–29% vs. 10%; *p* < 0.001).

The INSPIRE and COMMAND trials evaluated the use of risankizumab in UC [23,24]. The phase 3 INSPIRE induction trial included patients who had an intolerance or inadequate response to conventional therapy or to one or more advanced therapies. Importantly, no prior treatment with ustekinumab or another IL-23p19 inhibitor was allowed [25]. Patients were randomized to receive risankizumab IV 1200 mg or placebo every four weeks. Risankizumab treatment resulted in higher rates of clinical remission at week 12 (20.3% vs. 6.2%, *p*  < 0 .001), endoscopic improvement (36.5% vs. 12.1%, *p*  <  0.001) and combined histologic–endoscopic improvement (24.5% vs. 7.7%, *p*  <  0.001) compared to placebo. Patients who achieved response at week 12 were enrolled in the COMMAND trial, whereas non-responders were enrolled in an extended induction period of 12 weeks [25]. In the COMMAND maintenance trial, responding patients were re-randomized to receive risankizumab 180 mg or 360 mg SC every eight weeks or to an induction-only withdrawal control. At week 52, sustained clinical remission was significantly more frequent in both risankizumab treatment arms than in the withdrawal arm (180 mg: 40%; 360 mg: 38%; placebo: 25%). Risankizumab-treated patients also demonstrated higher rates of endoscopic improvement (51% and 48% vs. 32%; both *p* < 0.01) and combined histologic–endoscopic improvement (43% and 42% vs. 23%; both *p* < 0.01). To conclude, the described induction and maintenance therapies significantly improved clinical, endoscopic and histologic outcomes vs. placebo in a mixed population regardless of prior exposure to biologics [24,26].

#### 2.1.2. Guselkumab

Guselkumab was approved by the FDA for UC in 2024 and CD in 2025, with EMA-approvals obtained for both IBD types in 2025.

Guselkumab was evaluated for moderate-to-severe CD in the phase 2 GALAXI-1 trial, which incorporated both induction and maintenance phases [27]. During induction therapy, patients were randomized to receive guselkumab IV 200 mg, 600 mg or 1200 mg at weeks zero, four and eight; to receive ustekinumab IV ~6 mg/kilogram (kg) at week zero followed by SC 90 mg at week eight; or to receive an IV placebo. All guselkumab and ustekinumab treatment groups demonstrated significantly greater clinical response than placebo (all *p* < 0.05). Endoscopic response rates at week 12 were also superior for all guselkumab groups (200 mg: 37.7%; 600 mg: 36.5%; 1200 mg: 32.8%) and for ustekinumab (28.6%) compared with placebo (11.5%). However, endoscopic remission at week 12 was only significant in the guselkumab treatment arm with the highest dose (1200 mg: 16.0%) and in the ustekinumab group (14.3%), as compared with placebo (3.9%). In the maintenance phase, patients receiving guselkumab transitioned from IV to SC dosing [28]. By week 48, clinical remission rates exceeded 50% in all groups, demonstrating sustained efficacy (Table A1). Independent phase 3 GALAXI-2 and GALAXI-3 trials further confirmed both the short- and long-term efficacy of guselkumab versus placebo and showed statistical superiority over ustekinumab at week 48 [26].

The clinical efficacy of guselkumab for moderate-to-severe UC was assessed in the phase 2b and phase 3 QUASAR induction trials [29]. In the phase 2b study, patients were randomized to receive IV guselkumab 200 mg, IV guselkumab 400 mg, or placebo at weeks zero, four and eight. Both guselkumab regimens produced significantly higher week 12 clinical response rates (200 mg: 61.4%; 400 mg: 60.7%) compared with placebo (27.6%). Guselkumab-treated patients also demonstrated higher rates of endoscopic improvement, combined histologic–endoscopic improvement and endoscopic remission (*p* < 0.05, Table A1). In the subsequent phase 3 QUASAR induction trial, patients receiving guselkumab IV 200 mg at weeks zero, four and eight achieved significantly higher rates of clinical remission than placebo (22.6% vs. 7.9%, *p* < 0.001). Mirroring phase 2b findings, guselkumab was also associated with significantly greater rates of endoscopic improvement, combined histologic–endoscopic improvement and endoscopic normalization at week 12. Building on previous trials, patients who achieved an adequate response in phase 2b and phase 3 QUASAR trials were enrolled for the phase 3 maintenance trial [30]. Adequate response was defined as ≥30% baseline reduction, two point decrease in Mayo score, and rectal bleeding score ≤ 1 or ≥1 decrease. Patients who responded to induction therapy started with 200 mg SC every four weeks, 100 mg SC every eight weeks or IV placebo every four weeks. Placebo responders at week 24 switched to 200 mg SC guselkumab every four weeks. Placebo responders at week 12 induction therapy received an SC placebo every four weeks [30]. Findings demonstrated that both SC maintenance regimens, 200 mg every four weeks and 100 mg every eight weeks, were effective (50.0% and 45.2%, respectively), compared with placebo (18.9%, *p* < 0.001).

#### 2.1.3. Mirikizumab

Mirikizumab has been approved for UC (FDA and EMA, 2023) and for CD (FDA, 2025), with a definite approval expected in 2026.

For patients with CD, mirikizumab was examined in the phase 2 SERENITY induction and maintenance studies [31]. During induction, patients were randomized to receive mirikizumab IV at 200 mg, 600 mg, or 1000 mg, or a placebo, at weeks 0, 4 and 8. At week 12, patients treated with mirikizumab achieved significantly higher endoscopic response rates than those given the placebo, with rates of 37.5% for 600 mg and 43.8% for 1000 mg vs. 10.9% (*p* < 0.01). Endoscopic remission rates were likewise higher in the 600 mg and 1000 mg groups (15.6% and 20.3%; both *p* < 0.05) versus placebo (1.6%). Patients with at least a one-point reduction in SES-CD at week 12 were re-randomized to continue mirikizumab IV at their induction dose every four weeks or switched to 300 mg every four weeks. At week 52, endoscopic response (50% SES-CD reduction) was reached by 58.5% in the continued IV group and 58.7% in the SC group. The VIVID-1 trial is a phase 3, double-blind study evaluating mirikizumab in patients with CD who had failed at least one approved biologic or conventional therapy [32]. Participants were randomized to receive mirikizumab 900 mg intravenously at weeks zero, four and eight followed by 300 mg subcutaneously every 4 weeks from weeks 12 to 52, ustekinumab 6 mg/kg intravenously at week zero, followed by 90 mg subcutaneously every eight weeks from weeks eight to 52, or placebo. Endoscopic response occurred in 38% of patients treated with mirikizumab compared to 9% in the placebo group and overall adverse events were less frequent with mirikizumab than with placebo (*p* < 0.0001).

For patients with UC, the clinical efficacy of mirikizumab was first demonstrated in a phase 2 randomized controlled trial. In this study, patients received 50 mg, 200 mg, 600 mg mirikizumab IV or placebo at weeks zero, four and eight [33]. Clinical remission at week 12 was significantly higher than placebo for 200 mg (22.6%, *p* = 0.004). Remission rates for 50 mg (15.9%, *p* = 0.066) and 600 mg (11.5%, *p* = 0.142) did not differ significantly from placebo (4.8%). Endoscopic remission was independent of mirikizumab dose, with reported rates of 3.2% (50 mg), 3.2% (200 mg), 1.6% (600 mg), and 1.6% (placebo). In contrast, the phase 3 LUCENT-1 induction trial showed the superiority of mirikizumab IV 300 mg every four weeks over placebo for clinical remission (24.2% vs. 13.3%, *p* < 0.001) and endoscopic remission (36.3% vs. 21.1%, *p* < 0.001) at week 12 [34]. Moving to the maintenance phase, in the LUCENT-2 trial, patients responding to induction therapy were re-randomized to receive mirikizumab SC 200 mg or placebo every four weeks [34]. After 40 weeks, clinical remission remained significantly higher in the mirikizumab group (49.9%) compared with placebo (25.1%, *p* < 0.001). Endoscopic remission and combined histologic–endoscopic mucosal improvements were also significantly more frequent with mirikizumab. In the subsequent LUCENT-3 open-label extension trial, patients who achieved response or remission in LUCENT-2 continued mirikizumab SC 200 mg every four weeks through week 104 [35]. Patients in remission at week 52 exhibited numerically higher clinical remission rates at week 104 than responders (65.6% vs. 54.0%). Similarly, rates of endoscopic remission (77.3% vs. 65.3%) and combined histologic–endoscopic improvement as assessed by the Geboes histologic score (74.0% vs. 47.7%) were higher among week-52 remitters.

### 2.2. Oral IL-23 Pathway Inhibition

At present, all IL-23p19 inhibitors licensed for treatment of IBD are administered parenterally, using IV induction followed by SC maintenance regimens. Oral formulations, however, are generally more convenient and patient-friendly, aiming to establish localized and controlled drug concentrations at the site of inflammation in the intestines with generally favorable pharmacokinetic profiles [36]. The oral administration of monoclonal antibodies, such as the currently approved parenterally administered IL-23p19 inhibitors, is complicated by the esterase-rich and acidic environment of the gastrointestinal tract. Therefore, no oral formulation of IL-23p19 inhibitorshas currently advanced to clinical testing. However, there are different types of orally administered IL-23 pathway modulators currently being investigated in preclinical and clinical settings (Table 1). IL-23 inhibition by novel compounds may be achieved through direct antagonism of the IL-23 receptor or by inhibition of downstream IL-23-mediated signaling pathways, including JAK1 and TYK2. Current strategies focus on engineering protein-resistant antibody formats and developing non-antibody biologics, such as macrocyclic peptides and small molecules, to overcome the pharmacokinetic limitations of orally administered monoclonal antibodies while preserving pathway specificity. For example, an oral anti-IL-23 receptor single domain antibody, specifically engineered for protein resistance, was recently found to achieve IL-23 pathway inhibition in murine and non-human primate models [37]. Likewise, orally administered miniproteins that selectively bind to the IL-23 receptor and IL-17 receptor with antibody-like affinity reached the intestinal mucosa of murine models and significantly reduced inflammatory cytokines [38]. In parallel, oral bispecific inhibitors of TNF and IL-23 have also been in development. V56B2 is a novel bispecific domain antibody in which an IL-23p19-specific domain antibody is combined with a TNF-α-specific domain antibody [39]. V56B2 was found to reduce inflammatory phosphoprotein signatures in ex vivo intestinal biopsies of patients with UC. Similarly, SOR102 is an oral bispecific inhibitor of TNF and IL-23p19, which has been tested in a phase 1 trial involving patients with UC [40]. Pharmacokinetics (PKs) were consistent with predominantly localized gastrointestinal activity and minimal systemic absorption. In an additional small exploratory cohort of UC patients, SOR102 showed a dose- and frequency-dependent clinical response by day 42, with higher rates of symptomatic remission in the twice-daily SOR102 group compared with placebo [40].

Another subclass of IL-23 inhibitors is oral peptides specifically targeting IL-23 signaling. Oral PTG-200 (JNJ-67864238), a peptide targeting the IL-23 receptor, showed promising efficacy in preclinical studies [41]. However, in a phase 2 trial in patients with moderate to severe Crohn’s disease, predefined futility criteria were met, leading to early termination of the study. Icotrokinra is a different targeted oral macrocyclic peptide that selectively blocks IL-23 receptor activation [42,43]. In the ANTHEM-UC trial, a phase 2b study involving 252 adults with moderate-to-severe UC, preliminary results reported dose-dependent improvements in clinical response rates. At week 12 of once daily oral dosing of 400 mg icotrokinra, clinical remission rates were 30.2% compared to 11.1% in the placebo group, and 36.5% showed endoscopic improvement compared to 14.3% placebo [44]. Lasting clinical response rates were observed at week 28, with acceptable safety profiles [45]. Further trials involving icotrokinra have been initiated, concerning a phase 2b/3 trial in moderate-to-severe CD and a phase 3 trial in moderate-to-severe UC [46,47]. In addition, small-molecule inhibitors targeting JAK1 and TYK2, which act downstream of the IL-23 receptor, are currently under clinical development. Deucravacitinib is a selective, allosteric inhibitor of TYK2, an intracellular signaling mediator of IL-23. However, in three phase 2 clinical trials in patients with moderately to severely active CD and UC (LATTICE-CD, LATTICE-UC, and IM011-127), deucravacitinib demonstrated modest signals of efficacy, but failed to achieve the primary or secondary efficacy endpoints after 12 weeks of treatment across multiple dosing regimens [48]. These results are in line with another oral TYK2 inhibitor, VTX958, in which a phase 2 trial in patients with CD did not meet its primary endpoint of CDAI change from baseline [49]. Although inadequate dosing has been suggested as a potential explanation, this appears unlikely, as the doses tested exceeded those shown to be effective in psoriasis and included high-dose regimens that nonetheless failed to achieve primary endpoints, thereby raising the possibility that IL-23 pathway inhibition through downstream TYK2 inhibition may not represent an optimal therapeutic target in IBD [50]. Of interest, a next-generation allosteric TYK2 inhibitor, TAK-279 (zasocitinib), is currently in phase 2 evaluation for both UC and CD [51,52]. TAK-279 is designed for sustained 24 h pathway suppression and these trials will help understand if deeper TYK2 target engagement can translate to mucosal efficacy in IBD. Brepocitinib is an oral small molecule that selectively inhibits both TYK2 and JAK1, reducing downstream signaling of pro-inflammatory cytokines, including IL-12 and IL-23, and thereby potentially mimicking the dual pathway inhibition of agents such as ustekinumab. In phase 2b studies, brepocitinib has demonstrated significant improvements in disease activity compared with placebo in patients with moderate-to-severe UC [52]. At week 8 of treatment with 30 mg brepocitinib, the placebo-adjusted proportion of patients achieving clinical remission was 25.5%. Phase 2 data from the PIZZICATO umbrella induction trial indicated that brepocitinib also produced greater efficacy than placebo in moderate-to-severe CD, with favorable tolerability [53]. Brepocitinib 60 mg showed a significantly higher placebo-adjusted proportion of 21.4% of patients achieving endoscopic response. The brepocitinib clinical trial program in IBD has generated informative signals that support further investigation of TYK2/JAK1 dual inhibition.

## 3. IL-23p19 Class Effects

### 3.1. Head-to-Head Studies and Comparative Trials

Currently, no head-to-head randomized controlled trials comparing IL-23p19 inhibitors directly with each other have been published in IBD. Existing active comparator evidence is limited to studies in CD comparing IL-23p19 inhibitors against the IL-12p40/IL-23 inhibitor ustekinumab, in the absence of comparisons against anti-TNFs, vedolizumab, JAK-inhibitors or S1P modulators. Multiple phase 2 and 3 programs have now evaluated IL-23p19-inhibitors in CD using ustekinumab as an active comparator, warranting cautious extrapolation to IL-23p19-inhibitors in IBD. The SEQUENCE trial, a phase 3b controlled trial in patients with CD who experienced an inadequate response to anti-TNF therapy, randomized participants to risankizumab or ustekinumab for 48 weeks [54,55]. Risankizumab met the criterion for noninferiority to ustekinumab for clinical remission at week 24 (58.6% vs. 39.5%) and demonstrated superiority for endoscopic remission at week 48 (31.8% vs. 16.2%, *p* <0.001). Rates of adverse events were broadly comparable between the treatment arms. In the phase 3 GALAXI-2 and GALAXI-3 trials evaluating guselkumab in patients with CD, comparisons were made with both ustekinumab and placebo [26]. Pooled analyses demonstrated the statistically significant superiority of guselkumab over ustekinumab at week 48 for endoscopic response, endoscopic remission, and composite endpoints such as deep remission, while overall safety profiles remained broadly comparable. The VIVID-1 trial is a phase 3, double-blind study evaluating mirikizumab in patients with CD who had failed at least one approved biologic or conventional therapy [32]. Participants were randomized to receive mirikizumab 900 mg intravenously at weeks 0, 4 and 8 followed by 300 mg subcutaneously every 4 weeks from weeks 12 to 52, ustekinumab 6 mg/kg intravenously at week 0, followed by 90 mg subcutaneously every 8 weeks from weeks 8 to 52, or placebo. Endoscopic response occurred in 38% of patients treated with mirikizumab compared to 9% in the placebo group, and overall adverse events were less frequent with mirikizumab than with placebo. Preliminary results also suggest a trend toward higher endoscopic and clinical remission rates with mirikizumab than with ustekinumab [54]. The VEGA trial involved patients with UC, who were randomized into groups receiving guselkumab monotherapy and golimumab monotherapy or combination therapy with guselkumab and golimumab [56]. Combination therapy resulted in higher rates of clinical, endoscopic and histologic remission in UC compared with either monotherapy. The combination regimen produced early improvements, supporting the concept of dual IL-23 and TNF blockade. Safety outcomes were generally comparable across treatment arms, with no unexpected safety signals. Overall, the data for CD suggest that risankizumab and guselkumab are consistently at least non-inferior and frequently superior to ustekinumab, particularly with respect to objective endoscopic outcomes. Mirikizumab appears broadly comparable to ustekinumab, with numerically favorable results across several efficacy measures. We encourage physicians to take this into account when considering an IL-23p19-inhibitor for patients who were previously exposed to or failed to respond to ustekinumab. In other inflammatory conditions, such as in psoriasis, phase 3 comparative trials demonstrated that guselkumab achieved superior therapeutic efficacy compared with TNF-α inhibitor adalimumab [57].

### 3.2. Recent Meta-Analyses

Although no head-to-head trials have been conducted yet for IL-23p19 inhibitors in IBD, meta-analysis can be used for indirect comparisons between IL-23p19 inhibitors. However, it should be noted that these comparisons depend on assumptions of trial consistency that might not be fully met. Results should therefore be interpreted with caution. Recent systematic reviews and meta-analyses consistently show IL-23p19-inhibitors among the most effective non-TNF targeted therapies for moderate-to-severe IBD [58,59,60,61]. A recent meta-analysis evaluated advanced therapies for moderate-to-severe CD [62]. This supported the use of risankizumab, guselkumab and mirikizumab in biologic-naive patients and endorsed risankizumab and guselkumab for those who were previously exposed to biologics. Moderate-certainty evidence further favored risankizumab and guselkumab over vedolizumab and ustekinumab [62]. Another recent meta-analysis examined the efficacy of IL-23p19 and ustekinumab in moderate-to-severe CD. Guselkumab demonstrated the strongest induction efficacy among IL-23p19 inhibitors and placebo, although it was associated with the highest rate of adverse events during maintenance [63]. Risankizumab ranked highest for endoscopic outcomes and maintains a balanced efficacy–safety profile, while mirikizumab appears to offer an especially favorable balance between efficacy and serious adverse events. A meta-analysis that evaluated the efficacy of biologics and small molecules in CD ranked guselkumab as the second-highest for induction of clinical remission, surpassed only by infliximab combined with azathioprine [59]. Moreover, guselkumab and mirikizumab performed similarly well in maintaining clinical remission. For induction of endoscopic remission, risankizumab and guselkumab ranked comparably and were exceeded only by upadacitinib, whereas guselkumab was the top-ranked agent for maintaining endoscopic remission. An additional meta-analysis evaluating the efficacy of biologics and small molecules in UC showed that upadacitinib had the greatest efficacy in inducing clinical remission and endoscopic improvement, with risankizumab following second [64,65]. Risankizumab achieved the highest ranking for induction of histological remission, followed by guselkumab. For maintenance of histological remission, upadacitinib ranked first, again followed by guselkumab. A meta-analysis focusing on adverse events found no increase in overall adverse events versus placebo and even lower rates of serious adverse events, serious infections, and adverse event-related discontinuations in both CD and UC, while maintaining neutral infection and malignancy risks [60]. These recent comparative network meta-analyses consistently show that IL-23p19 inhibitors rank just below the most potent JAK inhibitors (particularly upadacitinib) in terms of efficacy for moderate-to-severe UC, while performing better or at least comparable to anti-TNF agents, vedolizumab and ustekinumab. Notably, IL-23p19-inhibitors appear to demonstrate a more favorable safety profile than JAK-inhibitors and S1P modulators. As for moderate-to-severe CD, IL-23p19 inhibitors appear efficient, although anti-TNF (especially the combination of infliximab and azathioprine) still shows the most favorable clinical outcomes [58,59,60,61]. As mentioned previously, physicians should take this into account when considering ustekinumab or an IL-23p19 inhibitor for patients with prior exposure to or inadequate response to anti-TNF.

## 4. Structural Characteristics of IL-23p19 Inhibitors

**Figure 4:**
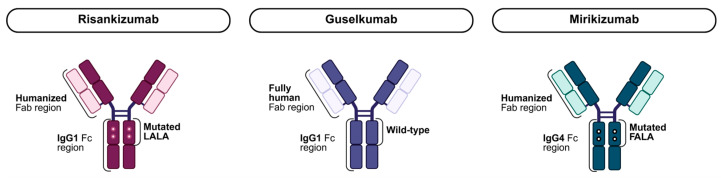
Structural features of IL-23p19 inhibitors approved for IBD. Risankizumab contains a humanized Fab region and an IgG1 Fc region incorporating a mutated LALA. Guselkumab has a fully human Fab region and an IgG1 wild-type Fc region. Mirikizumab contains a humanized Fab region and an IgG4 Fc region with a mutated FALA. Abbreviations: Fab, fragment antigen-binding; Fc, fragment crystallizable; FALA, phenylalanine to alanine and leucine to alanine substitutions; IBD, inflammatory bowel disease; IgG1, immunoglobulin G subclass 1; IL, interleukin; LALA, leucine to alanine substitutions.

Structural characteristics of IL-23p19 inhibitors may influence their therapeutic effects. Among these agents, risankizumab, guselkumab and mirikizumab differ in their structural properties, which may contribute to differences in cellular interactions and treatment responses (Figure 4). Risankizumab, a humanized IgG1 antibody, contains a leucine to alanine substitution at positions 234 and 235 (LALA), suppressing fragment crystallizable (Fc)-mediated functions [66,67]. Guselkumab, a fully human IgG1 antibody, contains a wild-type Fc-domain, whereas mirikizumab, a humanized IgG4 antibody, contains a phenylalanine to alanine substitution and leucine to alanine substitution at positions 234 and 235 (FALA) [67,68]. Since guselkumab has an unmodified IgG1 Fc- region, its Fc may interact with cluster of differentiation 64 (CD64) on myeloid cells [66,68]. Interestingly, one study demonstrated that guselkumab performs an Fc-mediated binding to CD64 on inflamed monocytes that subsequently reduces the release of IL-23 [69]. Thus, guselkumab not only neutralizes circulating IL-23 but also limits the production of IL-23. It would be of great interest to further study how this distinct Fc- domain contributes to different therapeutic outcomes between guselkumab on the one hand and risankizumab and mirikizumab on the other.

## 5. Pharmacokinetics and Dosing Considerations

### 5.1. Pharmacokinetics of IL-23p19 Inhibitors

Following absorption, distribution, metabolism and elimination, IL-23p19 inhibitors show linear PKs with a dose-proportional increase in exposure across IV and SC doses [70,71,72]. Peak plasma concentrations are achieved between three and 14 days after administration. Bioavailabilities vary substantially among the biologics, with reported bioavailabilities of 89% for risankizumab, 49% for guselkumab and 36.3% for mirikizumab. Risankizumab doses of 150 mg at week zero, four and every 12 weeks thereafter resulted in steady-state peak and trough plasma concentrations of 12 and 2 µg/mL, respectively [72]. Following SC administration of 100 mg guselkumab at week zero, four and every eight weeks thereafter, the steady-state trough serum concentration was approximately 1.2 µg/mL [71]. In phase 3 studies of psoriasis, risankizumab reached a steady-state volume of distribution of 11.4 ± 2.7 L (L) [72]. Following a single IV dose of guselkumab, the mean volume of distribution during the terminal phase in healthy subjects ranged between 7 and 10 L [71]. Patients with CD treated with mirikizumab showed a total volume of distribution (V_d_) of 4.40 L (14%), which was comparable with the total Vd of 4.83 L (21%) observed in patients with UC [70]. Risankizumab and mirikizumab are degraded in the same manner as endogenous IgG [70,72]. However, the metabolic pathway of guselkumab has not yet been characterized [71]. Clearance (CL) was 0.3 (±0.1) L/day in Phase 3 studies in patients with psoriasis treated with risankizumab, 0.288 to 0.479 L/day in healthy subjects who received guselkumab and 0.484 L/day in patients with CD or 0.549 L/day in patients with UC who received mirikizumab [70,71,72]. The mean terminal elimination half-life of risankizumab is approximately 28 to 29 days [72]. Patients with CD or UC treated with guselkumab showed a half-life (t_1/2_) of 17 days [71]. Reported t_1/2_ for mirkizumab in patients with CD or UC was 9.3 days [70]. Variations in the PK parameters mentioned above may arise due to heterogeneity among populations.

Risankizumab, guselkumab and mirikizumab demonstrated favorable immunogenicity profiles, with low rates of neutralizing antibodies observed in phase 2 and 3 clinical trials [70,71,72]. With regard to covariates influencing the pharmacokinetics of IL-23p19 inhibitors, body weight, gender and race did not clinically significantly impact the PK of risankizumab [72]. For guselkumab, it was found that body weight interferes with CL and V_d_ [71]. However, dose adjustments based on body weight are not warranted. Population (pop)PK analysis for mirikizumab demonstrated that the CL is not influenced by the concomitant use of immunomodulators [70].

### 5.2. Dosing Regimens for IV and SC Administration of IL-23p19 Inhibitors

**Figure 5:**
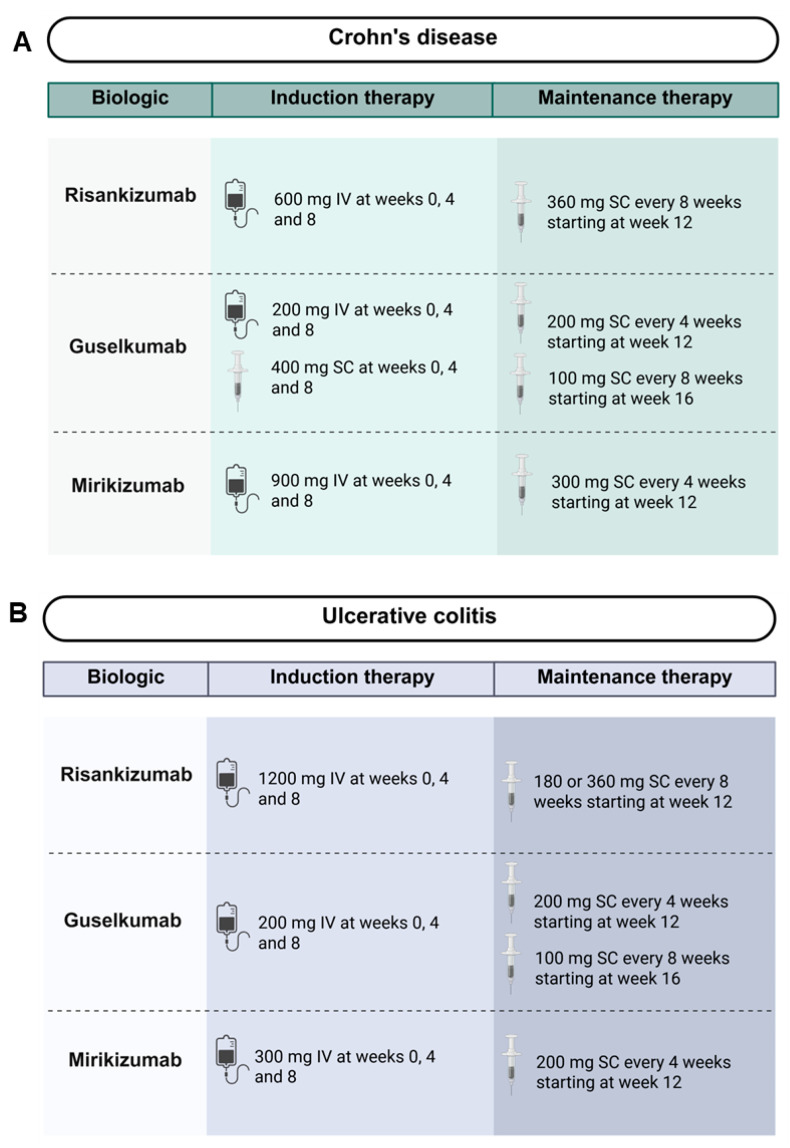
Overview of approved induction and maintenance regimens for risankizumab, guselkumab and mirikizumab for the treatment of IBD. (**A**) Recommended dosing regimens for patients with CD. (**B**) Recommended dosing regimens for patients with UC. Abbreviations: IBD, inflammatory bowel disease; IV, intravenous; mg, milligram; SC, subcutaneous.

Although risankizumab, guselkumab and mirikizumab are all IL-23p19 inhibitors, they differ in their dosing and administration schemes according to the FDA guidelines (Figure 5). During induction therapy for patients with CD and UC, all three biologics are administered at weeks zero, four and eight with varying administration routes and doses [73,74,75]. Risankizumab and mirikizumab are administered intravenously in both CD and UC [73,74]. Guselkumab is the only IL-23p19 inhibitor that can be administered either intravenously or subcutaneously during induction therapy for CD with comparable clinical and endoscopic remission rates [58,75]. Differences are also observed for maintenance therapy. Maintenance therapy for risankizumab and mirikizumab begins at week 12, whereas maintenance therapy for guselkumab can be initiated at week 12 or week 16 for both CD and UC [73,74,75]. Dosing interval for patients starting maintenance therapy at week 12 is every four weeks, whereas an eight-week interval is recommended for those starting at week 16 [75]. Moreover, risankizumab is administered every eight weeks, whereas mirikizumab is administered every four weeks [73,74].

### 5.3. Practical Dosing Considerations

For gastroenterologists, the selective choice of an IL-23p19 inhibitor is crucial in optimizing patient outcomes. Clinicians should evaluate multiple dimensions, including IBD phenotype, accessibility, adherence potential, patient preferences and treatment costs. Major barriers to adherence to IBD therapeutics are route of administration, frequency of administration and undesirable side effects [76]. Although many patients prefer IV administration over injections as a result of needle-related anxiety, one study demonstrated that 55% and 65% of patients favored SC over IV administration at four- and eight-week dosing intervals, respectively [77]. Risankizumab, guselkumab and mirikizumab all offer SC maintenance therapy with dosing intervals of four weeks for guselkumab and mirikizumab and dosing intervals of eight weeks for risankizumab and guselkumab, allowing treatment to be adapted to patient preferences. Another common factor that reduces medication adherence is side effects [77]. Overall, the safety profile of IL-23p19 inhibitors looks promising (Table 2), with most adverse events reported during clinical trials being mild to moderate in severity. Upper respiratory tract infections are the most frequently reported side effects for risankizumab and guselkumab, whereas injection site reactions are most commonly reported for mirikizumab [70,71,72]. Other frequently mentioned side effects include fatigue, headache, infections (upper respiratory and urinary tract infections), joint pain, and eczema [70,71,72]. Fortunately, anaphylaxis seems to be a rare side effect of IL-23p19 inhibitor use. Data on the use of IL-23p19 inhibitors in special populations is limited, as described later on. Moreover, IL-23p19 inhibitors show a more favorable side effect profile compared to TNF-α-inhibitors, reducing the risk of treatment discontinuation due to side effects. Despite these advantages, one major challenge of biologics is their high cost for patients and society. In psoriasis, risankizumab and guselkumab have demonstrated superior cost-effectiveness compared to other biologic therapies such as TNF-α inhibitors and ustekinumab [78,79]. Moreover, mirikizumab has shown greater cost-effectiveness than ustekinumab in UC [80]. These findings not only highlight the therapeutic efficacy of IL-23p19 inhibitors but also their economic viability.

## 6. Multi-Omics and Precision Medicine

**Figure 6:**
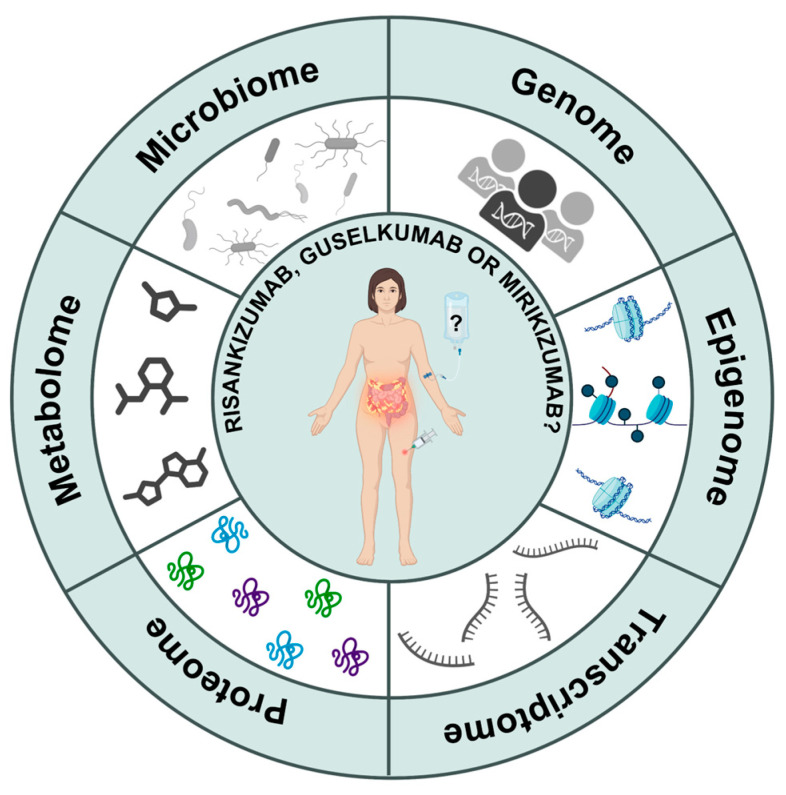
Multi-omics as a strategy to guide treatment with IL-23p19 inhibitors in IBD. At the center of the figure, a patient is shown initiating treatment with an IL-23p19 inhibitor. Surrounding the patient is a circle, representing six different multi-omics layers: genome, epigenome, transcriptome, proteome, metabolome and microbiome. These multi-omics should be considered for achieving the best treatment response in IBD. Abbreviations: IBD, inflammatory bowel disease; IL, interleukin.

Although IL-23p19 inhibitors show favorable efficacy profiles, a substantial portion of patients do not achieve remission [24,30,35,67]. Biomarkers are becoming increasingly important in predicting and evaluating treatment response in IBD and could contribute to optimizing treatment selection and outcomes [81]. Multi-omics approaches incorporate high-throughput analysis of multiple potential biomarkers to provide deeper insights into disease mechanisms, potentially bridging the gap between biomarkers and individual patients. A multi-omics strategy, which includes, among others, genomics, epigenomics, transcriptomics, proteomics, metabolomics and gut microbiome profiling, enabling a systems-level understanding of IBD pathophysiology that cannot be achieved by profiling one single type of biologic data [82]. Furthermore, multi-omics research facilitates the identification of new unrecognized biomarkers that can potentially be applied clinically for various utilities, including diagnosis, monitoring of disease activity, and predicting and evaluating treatment outcomes. Furthermore, multi-omics characterization can potentially be used to predict the treatment response to a certain IL-23p19 inhibitor, which is already demonstrated in TNF-α inhibitors, vedolizumab, and ustekinumab (Figure 6) [81,83]. Despite these issues, multi-omics studies in relation to IL-23p19 inhibition have already revealed some interesting insights, as outlined below. Due to the limited availability of IL-23p19 inhibition in IBD, findings in other immune-mediated inflammatory disorders (IMIDs) and other biologics such as ustekinumab are occasionally discussed. However, these findings should be interpreted as hypothesis-generating, rather than predictive of therapeutic outcomes in IBD.

### 6.1. Current Knowledge on Different Multi-Omics Domains

#### 6.1.1. Genomics

To date, only limited research has been conducted on genetic polymorphisms in association with response to IL-23p19 inhibitors in IBD, but some promising results have been demonstrated for TNF-α inhibitors and ustekinumab. For example, one study demonstrated that a pharmacogenetic profile or passport based on genetic data from thiopurine and or TNF-α inhibitor users accurately predicted adverse effects in 36% of the cases [84]. Another study reported a single polymorphism in the PTPN2 rs7234029 risk allele in CD, which is associated with nonresponse to ustekinumab [85]. They hypothesized that PTPN2 rs7234029 may alter binding sites for various transcription factors and therefore modulate inflammatory signaling. Although further research is warranted, these findings suggest that pharmacogenetic profiles could meaningfully reduce preventable adverse drug reactions. Still, most of our current understanding of genetic polymorphisms in treatment response comes from small, single-center retrospective studies, typically limited to homogeneous populations and focused on single-nucleotide polymorphisms [86].

#### 6.1.2. Epigenomics

Epigenetics refers to reversible modifications to the deoxyribonucleic acid (DNA), such as DNA methylation, histone modulation and chromatin remodeling, that regulate gene expression [87]. Epigenetic states are dynamic and influenced by diet, microbial composition, inflammatory state and therapeutic interventions, leading to changes throughout a patient’s life [88]. Such fluctuations offer a potential window for positive therapeutic influences. One landmark study (EPIC-CD) looked into peripheral blood DNA methylation signatures to predict adalimumab, vedolizumab and ustekinumab response in patients with IBD [89]. Their findings suggest that DNA methylation could help in identifying responders and non-responders before treatment with vedolizumab or ustekinumab. It would therefore be of interest to extend such epigenomic studies to IL-23p19 inhibitors to explore the role of epigenomics in predicting treatment response to IL-23p19 inhibitor therapy.

#### 6.1.3. Transcriptomics

Examining transcriptomic changes may provide valuable insights for researchers and clinicians into how therapeutic interventions alter gene activity. Risankizumab treatment was shown to reduce the expression of key inflammatory genes in ileal and colonic tissue associated with CD activity, including S100 calcium-binding protein A8 (S100A8), S100 calcium-binding protein A9 (S100A9), interleukin-8 (IL8), matrix metallopeptidase 1 (MMP1), IFN-γ, lipocalin-2 (LCN2), tissue inhibitor of metalloproteinases 1, TNF and STAT3 [84]. Even larger reductions in the expression of S100 calcium-binding protein A12 and MMP3 in the colon were observed [84]. Kirsten rat sarcoma viral oncogene homolog activation induced upregulation of several cytokine signaling genes, including IL-17, IL-6, IFN-γ, IL-12 and the JAK/STAT3 pathway, in both the ileum and colon [84]. Additionally, TNF-α signaling was elevated in response to NFκB activation [84]. Regarding guselkumab, one study showed that guselkumab induces downregulation of neutrophil, monocyte, eosinophil and macrophage gene sets and upregulation of B cell, T cell and NK cell gene sets in psoriatic arthritis (PsA) [90]. Another study in psoriasis using data from the VOYAGE-1 trial showed that the expression of IL-23/IL-17 pathway–associated and psoriasis-associated genes was reduced with guselkumab treatment. Measuring the expression of key inflammatory genes in blood can help evaluate treatment response, not only in psoriasis and active PsA, but also in IBD.

#### 6.1.4. Proteomics

Proteomics provides a powerful tool to predict and evaluate treatment response. For infliximab, several interesting proteomic results have already been published regarding the prediction of treatment response [91]. Although proteomic data on IL-23p19 inhibitors in IBD are sparse, some interesting results have been published already. One study identified IL-23–dependent proteins in IBD mouse models using shotgun proteomic analysis [92]. S100A8, S100A9, regenerating protein 3b (REG3b), REG3c, and LCN2, which are deleted in malignant tumor 1 and macrophage migration inhibitory factor (MIF), showed associations with disease severity and dose-dependent inhibition of the IL-23 receptor or IL-23(p19) by monoclonal antibodies. Subsequently, these biomarkers were tested in a validation model, where all, except DMBT1, were confirmed. Interestingly, in patients with CD, serum levels of S100A8/A9, MIF, pancreatitis-associated protein (human homolog of REG3b/c), LCN2 and C-C motif chemokine ligand (CCL20) were increased compared to controls. All except MIF and CCL20 were also elevated in fecal samples, demonstrating further clinical relevance of these findings. Altogether, these findings highlight the relevance of taking downstream IL-23 protein biomarkers into account when evaluating the efficacy of IL-23p19 inhibitor therapy.

#### 6.1.5. Microbiome and Metabolomics

The gut microbiome represents another key layer of biological data that can be leveraged for predicting treatment response. For TNF-α inhibitors, vedolizumab and ustekinumab, correlations between gut microbiome composition and treatment outcomes have been explored extensively [93,94,95,96]. For example, one study on changes in the gut microbiome in UC demonstrated a decreased abundance of *Burkholderia–Caballeronia–Paraburkholderia* and *Staphylococcus*, alongside an increased abundance of *Bifidobacterium* and Dorea in patients treated with adalimumab who achieved remission [95]. Another study showed that patients with CD who responded to ustekinumab exhibit higher microbial community α-diversity and greater abundance of *Roseburia inulinivorans* and a *Burkholderia* species at week 14 compared to baseline [93]. Patients who responded to ustekinumab also demonstrated enrichment of microbial pathways, including branched-chain amino acid synthesis pathways. Moreover, a systematic review described that patients with IBD who achieved a response to vedolizumab therapy had higher levels of specific bacteria, including *Faecaliumbacterium prausnitzii* and *Anaerostipes hadrus*, producing butyrate [95]. Directly following microbiome profiling, metabolomics can be used to study the metabolites produced by the microbiome. However, to date, no IL-23p19 inhibitor-specific studies on gut microbiome or metabolomics have been performed. Considering the insights gained from other biologics, expanding research in this field would be of great interest and would help complete the multi-omics profile of IL-23p19 inhibitors in IBD.

### 6.2. Therapeutic Drug Monitoring and Model-Informed Precision Dosing: Bridging the Gap Between Multi-Omics and Treatment Algorithms

Treatment strategies such as proactive therapeutic drug monitoring (TDM) and model-informed precision dosing (MIPD) are modern approaches to personalize treatments that can be informed by multi-omics data. Proactive TDM measures serum drug levels and adjusts treatment regimens regardless of disease activity, whereas reactive TDM responds to clinical, biochemical or endoscopic disease activity. Proactive TDM has already shown to be superior to reactive TDM for infliximab and ustekinumab [97,98]. Nevertheless, proactive TDM is still in its early stages for IL-23p19 inhibitors. To date, only one phase 4 clinical trial is studying TDM for risankizumab in patients with psoriasis [99]. However, the study is still recruiting, and no results have been published yet. To expand on new treatment approaches, another emerging strategy is MIPD. MIPD uses Bayesian statistics to combine population PK data with patient-specific data in order to generate individualized dosing regimens. In this way, dashboard-driven dosing can be used to achieve patient-specific therapeutic targets. This approach has been studied increasingly for infliximab in IBD with promising results [100,101]. MIPD has not been studied yet for IL-23p19 inhibitors in IBD. Recently, a precision-dosing dashboard was successfully developed for risankizumab in psoriasis, paving the way for future research on MIPD in IBD as well as other IL-23p19 inhibitors [102]. It would be of great interest to incorporate currently identified and emerging biomarkers from multi-omics studies into TDM and MIPD. In this way, biomarker data can be integrated into treatment algorithms and can subsequently be used in precision medicine. Researchers and clinicians should take into account that biomarkers relevant to treatment response may differ between patients.

## 7. Future Perspectives

Beyond the efficacy demonstrated in clinical trials, important knowledge gaps remain, hindering the optimal introduction of IL-23p19 inhibitors in clinical practice. These include the lack of real-world data and limited insights into treatment outcomes in IBD-specific subtypes. Again, because data on IL-23p19 inhibition in IBD remain limited, findings on other IMIDs and other biologics such as ustekinumab are occasionally discussed.

### 7.1. Real-World Data on IL-23p19 Inhibitors

Real-world data from a GETAID study assessing the long-term outcomes of risankizumab in CD demonstrated steroid-free clinical remission rates of approximately 46% at week 26 and 52 [103]. Clinical remission rates were 52% and 48% at weeks 26 and 52, respectively [103]. In a large retrospective multicenter study on risankizumab in CD, a week 12 clinical remission rate of 49.7% was reached [104]. Cumulative rates of clinical and endoscopic remission at week 52 were 65.0% and 49.5%, respectively [104]. Furthermore, a real-world comparative study showed superior effectiveness of risankizumab over ustekinumab at week 8 and 28 [105]. No real-world effectiveness studies have been published for guselkumab in IBD. However, one multicenter study studied the 5-year efficacy of guselkumab in psoriasis and demonstrated sustained long-term effectiveness of guselkumab for patients with psoriasis, highlighting its potential relevance for further studies in IBD [106]. Currently, the GORGEOUS trial is evaluating the real-world performance of guselkumab in IBD [107]. Mirikizumab achieved clinical remission at week 12 for UC in real-world studies of 42% [108,109]. A study that focused on patients with therapy-refractory UC reported a clinical remission rate of 67.3% at week 12 [110]. Collectively, these studies show real-world data demonstrating that risankizumab is well-tolerated in CD, mirikizumab in UC and that further research on real-world data is warranted, especially for guselkumab.

### 7.2. IBD-Specific Subpopulations

Although IL-23p19 inhibitors have shown promising results, their efficacy and applicability across diverse IBD subpopulations remain underexplored. Patients with perianal CD represent a difficult-to-treat subgroup for whom the applicability of IL-23p19 inhibitors has not been studied extensively. A single case report describing risankizumab use in a patient with recurrent isolated perianal CD suggests the potential efficacy of risankizumab in this subgroup [111]. Given the therapeutic complexity of perianal CD, evaluating the safety and efficacy of IL-23p19 inhibitors in this subgroup would be of great interest. Building on this preliminary evidence, the ongoing phase 3 FUZION trial assesses the potential use of guselkumab for fistulizing perianal CD [112]. A post hoc subanalysis, combining data from the phase 3 ADVANCE, MOTIVATE and FORTIFY studies, demonstrated fistula closure for 28.6% of Asian patients at induction therapy and 57.1% of Asian patients for maintenance therapy using Risankizumab [113]. Another challenging subgroup includes patients with refractory CD or UC. Preliminary data on risankizumab and mirikizumab suggest that these biologics are safe and effective for inducing and maintaining remission in refractory CD and UC, respectively [114,115]. The applicability of IL-23p19 inhibitors in pediatric CD and UC is increasingly being studied, with multiple ongoing clinical trials [114,116,117,118]. The SHINE-1 trial demonstrated that mirikizumab therapy in pediatric patients with UC resulted in clinical improvement when looking at clinical and endoscopic remission scores at week 12 and week 52, with a favorable safety profile [119]. Moreover, mirikizumab also appeared to be effective for pediatric patients with refractory disease. These results point to a potential clinical benefit of IL-23p19 inhibitors in pediatric patients with IBD and provide a strong rationale for future research in this subgroup. Unfortunately, data on the safety of IL-23p19 inhibitors throughout pregnancy remain limited. One systematic review assessing the use of biologics during pregnancy demonstrated that physiological and anatomical changes throughout pregnancy may influence the PK of biologics [120]. However, they did not specifically assess IL-23p19 inhibitors. Available clinical data on drug-associated risk of major birth defects, miscarriage or other adverse maternal or fetal outcomes are insufficient to provide evidence-based recommendations regarding the use of IL-23p19 inhibitors throughout pregnancy [73,74,75]. An early report from the Pregnancy in IBD and Neonatal Outcomes (PIANO) registry on three patients with CD showed that low concentrations of risankizumab are transferred to the infant [121]. One recent study on the use of risankizumab during pregnancy in humans with IBD suggests that risankizumab does not appear to increase the risk of adverse outcomes, such as preterm birth, low birth weight, cesarean delivery, congenital anomalies and other neonatal complications, compared to patients treated with anti-TNF agents or conventional therapy [122]. Furthermore, most knowledge on IL-23p19 inhibitors in pregnancy stems from animal studies. Animal studies in cynomolgus monkeys have already shown no infant toxicity related to IL-23p19 inhibitors [73,74,75]. Adult female cynomolgus monkeys and all infants from risankizumab- and mirikizumab-treated groups had measurable serum concentrations up to 91 and 28 days postpartum, respectively [73,74]. Adult female cynomolgus monkeys administered 50 mg/kg risankizumab showed increased fetal and infant loss compared to the vehicle group [75]. To conclude, limited information on IL-23p19 inhibitor use in humans throughout pregnancy is available, limiting current conclusions. Given that pregnancy is a common concern for patients with IBD, additional research is warranted in order to meet this unmet clinical need.

### 7.3. Defining the Therapeutic Role of IL-23p19 Inhibitors in the IBD Treatment Landscape

Not only should the efficacy of these treatments be studied more extensively across IBD-specific subtypes, but these insights are also crucial for defining the therapeutic positioning of IL-23p19 inhibitors. The number of biologic therapies has more than doubled over the past decades [123]. Most of our current knowledge stems from initial biologics, such as infliximab and adalimumab. Hence, studying the position of emerging biologics in order to distinguish them from other biologic therapies is increasingly important [123]. In addition, current treatments for IBD appear to be reaching a ‘therapeutic ceiling’. One possible solution to overcome this issue is to alter the order of biologic administration or combine different advanced therapies [124]. Consequently, as mentioned previously, the VEGA and SEQUANCE trials have already focused on this. They have shown that a combination of guselkumab with golimumab is especially relevant for patients with UC who have failed to respond to TNF-α-inhibitors. Moreover, risankizumab showed superiority over ustekinumab in achieving steroid-free clinical and endoscopic remission with lower rates of reported serious adverse events, which is promising for the clinical efficacy and safety of risankizumab [55]. Other ongoing trials, including DUET-CD and DUET-UC, are investigating the combination of guselkumab with golimumab in moderately-to-severely active CD or UC [125,126]. Results from these and future trials can be used to optimize treatment algorithms and support the combination of existing therapies. Unfortunately, the therapeutic position of mirikizumab, as well as its option for combination therapy, has not been studied yet, necessitating further investigation in the coming years.

## 8. Concluding Remarks

IL-23p19 inhibitors have demonstrated favorable efficacy, safety and tolerability profiles in phase 3 clinical trials. Moreover, IL-23p19 inhibitors have shown to be comparable or even superior to ustekinumab in inducing clinical and endoscopic remission in CD, with comparable safety profiles observed. Direct comparative studies of IL-23p19 inhibitors are currently lacking, and research on their use in IBD-specific subpopulations remains limited. Since they are not based on head-to-head trials, most comparisons between IL-23p19 inhibitors should be interpreted with caution. Unfortunately, despite promising results, a substantial portion of patients continues to experience disease symptoms. Future research should therefore prioritize the identification of multi-omics-driven biomarkers and their relation to IL-23p19 inhibitor response. This knowledge can then be integrated into TDM and MIPD approaches, facilitating precision medicine for IL-23p19 inhibitor use in IBD.

## Figures and Tables

**Table 1:** Overview of oral formulations of IL-23-p19 inhibitors. Abbreviations: IL, interleukin; JAK1, janus kinase one inhibitor; TNF, tumor necrosis factor; TYK2, tyrosine kinase two.

Name	Targeting Mechanism
V56B2	Neutralizing TNF-α domain and IL-23p19 domain.
SOR102	Neutralizing TNF-α domain and IL-23p19 domain.
PTG-200	Peptide targeting the IL-23 receptor.
VTX958	TYK2 inhibitor.
Icotrokinra	Macrocyclic peptide that selectively blocks IL-23 receptor activation.
Deucravacitinib	TYK2 inhibitor.
Zasocitinib	TYK2 inhibitor.
Brepocitinib	TYK2 and JAK1 inhibitor.

**Table 2:** Overview of reported side effects for IL-23p19 inhibitors in IBD.

Side Effects	Risankizumab [72]	Guselkumab [71]	Mirikizumab [70]
Very common (more than 1 in 10)	Upper respiratory infections.	Upper respiratory infections.	Injection site reactions.
Common (up to 1 in 10)	Fatigue, headache, fungal skin infection, injection site reactions, itching, rash and eczema.	Headache, injection site reactions, skin rash, joint pain, diarrhea and increased liver enzymes.	Headache, upper respiratory tract infections, rash and joint pain.
Uncommon (1 in 100)	Small raised red bumps on the skin and urticaria.	Neutropenia, herpes simplex infection, fungal infection of the skin, gastroenteritis and urticaria.	Shingles, itching, urticaria, increased liver enzymes.
Rare (1 in 1000)	Anaphylaxis.	Anaphylaxis.	Anaphylaxis.

## Data Availability

No new data were created or analyzed in this study. Data sharing is not applicable to this article.

## Abbreviations

The following abbreviations are used in this manuscript:

BLIMP-1	B lymphocyte-induced maturation protein 1
CDAI	Crohn’s Disease Activity Index
CD	Crohn’s disease
CD	Cluster of differentiation
CCL	C-C motif chemokine ligand
CL	Clearance
DC	Dendritic cell
EMA	European Medicines Agency
FALA	Phenylalanine to alanine substitution and leucine to alanine substitution
Fc	Fragment crystallizable
FDA	Food and Drug Administration
Fig.	Figure
GATA3	GATA-binding protein 3
IBD	Inflammatory bowel disease
IκBα	Inhibitor of nuclear factor-κB alpha
Ig	Immunoglobulin
IL	Interleukin
IV	Intravenous
JAK2	Janus kinase 2
Kg	Kilogram
L	Liters
LALA	Leucine to alanine substitution
LCN-2	Lipocalin-2
MIF	Macrophage migration inhibitory factor
MIPD	Model-informed precision dosing
ML	Milliliters
Mg	Milligram
MMP	Matrix metallopeptidase
NF-κB	Nuclear factor κ-light-chain-enhancer of activated B cells
NK	Natural killer
PK	Pharmacokinetics
Pop	Population
PsA	Psoriatic arthritis
RANKL	Receptor activator of nuclear factor-κB ligand
REG	Regenerating protein
RORγt	RAR-related orphan receptor γ t
Satb1	Special AT-rich sequence-binding protein 1
SC	Subcutaneous
SES-CD	Simple Endoscopic Score for Crohn’s Disease
STAT	Signal transducers and activators of transcription
t_1/2_	Half life
TDM	Therapeutic drug monitoring
Th17 cells	T-helper 17 cells
TYK2	Tyrosine kinase 2
UC	Ulcerative colitis
V_d_	Volume of distribution

## Appendix A

**Table A1:** An overview of completed and ongoing clinical trials, including their primary, endoscopic and/or histologic outcomes, evaluating IL-23p19 inhibitors for the treatment of IBD. Abbreviations: CD, Crohn’s disease; IBD, inflammatory bowel disease; CDAI, Crohn’s Disease Activity Index; IL, interleukin; IV, intravenous; mg, milligrams; NR, not reported; q, every; SC, subcutaneous; SES-CD, Simple Endoscopic Score for Crohn’s Disease; UC, ulcerative colitis; w, week; wk, weeks.

Trial Name, Reference	Study Population	Study Arms, *N*	Clinical Remission	Endoscopic Response/ Remission	Histological Response
**Risankizumab (Skyrizi)**					
Feagan et al., 2017 [20] Phase 2 RCT Induction	CD CDAI 220–450 and CDEIS ≥ 7 or ≥4 for L1 disease Previous anti-TNF and vedo allowed	1: Risankizumab IV 200 mg q4w (*n* = 41) 2: Risankizumab IV 600 mg q4w (*n* = 41) 3: Placebo (*n* = 39)	Clinical remission (CDAI score < 150) at wk 12 - Risankizumab 200 mg 24% (*p* = 0.31 vs. placebo) - Risankizumab 600 mg 37% (*p* = 0.03 vs. placebo) - Placebo: 15%	Endoscopic response (>50% reduction in CDEIS) at wk 12: - Risankizumab 200 mg 27% (*p* = 0.10 vs. placebo) - Risankizumab 600 mg 37% (*p* = 0.01 vs. placebo) - Placebo: 13% Endoscopic remission (CDEIS score ≤ 4 or ≤2 in L1 disease) at wk 12: - Risankizumab 200 mg 15% (*p* = 0.04 vs. placebo) - Risankizumab 600 mg 20% (*p* = 0.01 vs. placebo) - Placebo: 3%	Mucosal healing (absence of mucosal ulceration) - Risankizumab 200 mg 2% (*p* = 0.97 vs. placebo) - Risankizumab 600 mg 7% (*p* = 0.31 vs. placebo) - Placebo: 3%
ADVANCE D’Haens et al., 2022 [21] Phase 3 RCT induction	CD CDAI 220–450 and SES-CD ≥6, or ≥4 for L1 disease Intolerance or inadequate response to conventional and/or biologic therapies	1: Risankizumab IV 600 mg q4w (*n* = 336) 2: Risankizumab 1200 mg q4w (*n* = 339) 3: Placebo (*n* = 175)	Clinical remission (CDAI score < 150) at wk 12 - Risankizumab 600 mg 45% (*p <* 0.001 vs. placebo) - Risankizumab 1200 mg 42% (*p <* 0.001 vs. placebo) - Placebo: 25%	Endoscopic response (SES-CD decrease of >50% from baseline; or ≤2 point deduction in L1 disease) at wk 12 - Risankizumab 600 mg 40% (*p <* 0.001 vs. placebo) - Risankizumab 1200 mg 32% (*p <* 0.001 vs. placebo) - Placebo: 28%	NR.
MOTIVATE D’Haens et al., 2022 [21] Phase 3 RCT induction	CD CDAI 220–450 and SES-CD ≥6, or ≥4 for L1 disease only patients with inadequate response to biologic therapies	1: Risankizumab IV 600 mg q4w (*n* = 191) 2: Risankizumab 1200 mg q4w (*n* = 191) 3: Placebo (*n* = 187)	Clinical remission (CDAI score < 150) at wk 12 - Risankizumab 600 mg: 42% (*p* < 0.001 vs. placebo) - Risankizumab 1200 mg: 40% (*p* < 0.001 vs. placebo) - Placebo: 17%	Endoscopic response (SES-CD decrease of >50% from baseline; or ≤2 point deduction in L1 disease) at wk 12: - Risankizumab 600 mg: 29% (*p* < 0.001 vs. placebo) - Risankizumab 1200 mg: 34% (*p* < 0.001 vs. placebo) - Placebo: 11%	NR
FORTIFY Ferrante et al., 2022 [22] Phase 3 RCT maintenance	CD Participants in ADVANCE or MOTIVATE trial achieving clinical response to risankizumab (≥30% decrease in mean stool frequency or ≥30% decrease in mean daily abdominal pain score)	1: Risankizumab SC 180 mg q8w (*n* = 157) 2: Risankizumab SC 360 mg q8w (*n* = 141) 3: Placebo q8w (*n* = 164)	Clinical remission (CDAI score < 150) at wk 52 - Risankizumab 180 mg: 55% (*p* < 0.01 vs. placebo) - Risankizumab 360 mg: 52% (*p* < 0.01 vs. placebo) - Placebo: 41%	Endoscopic response (SES-CD decrease of >50% from baseline of the induction study; or ≤2 point deduction in L1 disease) at wk 52: - Risankizumab 180 mg: 47% (*p* < 0.001 vs. placebo) - Risankizumab 360 mg: 47% (*p* < 0.001 vs. placebo) - Placebo: 22% Endoscopic remission (SES-CD ≤ 4 and ≥2-point reduction with no single subscore > 1) at wk 52 - Risankizumab 180 mg: 30% (*p* < 0.01 vs. placebo) - Risankizumab 360 mg: 39% (*p* < 0.01 vs. placebo) - Placebo: 13%	NR
SEQUENCE Peyrin-Biroulet et al., 2024 [55] Phase 3B RCT	CD CDAI 220–450 and SES-CD ≥6, or ≥4 for L1 disease Patients with inadequate response to biologic therapies	1: Risankizumab 600 mg IV induction at weeks 0, 4, and 8, followed by a 360 mg SC maintenance q8w (*n* = 255) 2: Ustekinumab weight-based IV induction at weeks 0, followed by a 90 mg SC maintenance q8w (*n* = 265)	Clinical remission (CDAI score < 150) at wk 48 - Risankizumab: 61% (*p* < 0.001 vs. ustek) - Ustekinumab: 41%	Endoscopic response (SES-CD decrease of >50% from baseline; or ≤2 point deduction in L1 disease) at wk 48 - Risankizumab: 45% (*p* < 0.001 vs. ustek) - Ustekinumab: 22% Endoscopic remission (SES-CD ≤ 4 and ≥2-point reduction with no single subscore > 1) at wk 48 - Risankizumab: 32% (*p* < 0.001 vs. ustek) - Ustekinumab: 16%	NR
INSPIRE Louis et al., 2024 [24] Phase 3 RCT induction	UC Adapted mayo score 5–9 points and Endoscopic subscore of 2–3 Intolerance or inadequate response to conventional and/or biologic therapies, with exclusion of ustek and other Il23p19-inhibitors	1: Risankizumab IV 1200 mg q4w (*n* = 652) 2: Placebo q4w (*n* = 325)	Clinical remission (≥1-point decrease in stool frequency subscore or subscore 0 or 1, and rectal bleeding subscore of 0, and endoscopic subscore 0 or 1) at wk 12: - Risankizumab 1200 mg: 20% (*p* < 0.001 vs. placebo) - Placebo: 6%	Endoscopic response (Mayo endoscopic subscore 0 or 1) at wk 12: - Risankizumab 1200 mg: 37% (*p* < 0.001 vs. placebo) - Placebo: 12% Endoscopic remission (Mayo endoscopic subscore of 0) at wk 12: - Risankizumab 1200 mg: 11% (*p* < 0.001 vs. placebo) - Placebo: 3%	Histologic improvement (Mayo endoscopic subscore 0 or 1 and Geboes score < 3.2) at wk 12: - Risankizumab 1200 mg: 25% (*p* < 0.001 vs. placebo) - Placebo: 8% Histological remission (Mayo endoscopic subscore of 0 and Geboes score < 2.1) at wk 12: - Risankizumab 1200 mg: 6% (*p* < 0.001 vs. placebo) - Placebo: 1%
COMMAND Louis et al., 2024 [24] Phase 3 RCT maintenance	UC Participants in INSPIRE trial achieving adequate response to risankizumab (reduction in adapted mayo score ≥ 30% from baseline and a decrease of ≥1 in rectal bleeding score or an absolute rectal bleeding score ≤ 1)	1: Risankizumab SC 180 mg q8w (*n* = 193) 2: Risankizumab SC 360 mg q8w (*n* = 195) 3: Placebo (*n* = 196)	Clinical remission (≥1-point decrease in stool frequency subscore or subscore 0 or 1, and rectal bleeding subscore of 0, and endoscopic subscore 0 or 1) at wk 52: - Risankizumab 180 mg: 40% (*p* < 0.001 vs. placebo) - Risankizumab 360 mg: 38% (*p* < 0.001 vs. placebo) - Placebo: 25%	Endoscopic response (Mayo endoscopic subscore 0 or 1) at wk 52: - Risankizumab 180 mg: 51% (*p* < 0.01 vs. placebo) - Risankizumab 360 mg: 48% (*p* < 0.01 vs. placebo) - Placebo: 32% Endoscopic resmission (Mayo endoscopic subscore 0) at wk 52: - Risankizumab 180 mg: 23% (*p* = 0.01 vs. placebo) - Risankizumab 360 mg: 24% (*p* = 0.01 vs. placebo) - Placebo: 15%	Histologic improvement (Mayo endoscopic subscore 0 or 1 and Geboes score < 3.2) at wk 52: - Risankizumab 180 mg: 43% (*p* < 0.01 vs. placebo) - Risankizumab 360 mg: 42% (*p* < 0.01 vs. placebo) - Placebo: 24% Histological remission (Mayo endoscopic subscore of 0 and Geboes score < 2.1) at wk 52: - Risankizumab 180 mg: 13% (*p* = 0.21 vs. placebo) - Risankizumab 360 mg: 16% (*p* = 0.06 vs. placebo) - Placebo: 10%
**Guselkumab (Tremfya)**					
GALAXI-1 Sandborn et al., 2022 [27] Phase 2 RCT induction	CD CDAI 220–450 and SES-CD ≥3 Inadequate response or intolerance to conventional therapy or biologic therapy	1: Guselkumab IV 200 mg q4w (*n* = 61) 2: Guselkumab IV 600 mg q4w (*n* = 63) 3: Guselkumab IV 1200 mg q4w (*n* = 61) 4: Ustekinumab IV ∼6 mg/kg at wk 0 and SC 90 mg at wk 8 (*n* = 63) 5: Placebo (*n* = 61)	Clinical remission (CDAI score < 150) at wk 12: - Guselkumab 200 mg: 57% (*p* < 0.05 vs. placebo) - Guselkumab 600 mg: 56% (*p* < 0.05 vs. placebo) - Guselkumab 1200 mg: 46% (*p* < 0.05 vs. placebo) - Ustekinumab: 46% (*p* < 0.05 vs. placebo) - Placebo: 16%	Endoscopic response (≥50% improvement in SES-CD or SES-CD ≤ 2) at wk 12 - Guselkumab 200 mg: 38% (*p* < 0.05 vs. placebo) - Guselkumab 600 mg: 37% (*p* < 0.05 vs. placebo) - Guselkumab 1200 mg: 33% (*p* < 0.05 vs. placebo) - Ustekinumab: 29% (*p* < 0.05 vs. placebo) - Placebo: 12% Endoscopic remission (SES-CD ≤ 2) at wk 12 - Guselkumab 200 mg: 16% (*p* = 0.064 vs. placebo) - Guselkumab 600 mg: 10% (*p* = 0.255 vs. placebo) - Guselkumab 1200 mg: 16% (*p* < 0.05 vs. placebo) - Ustekinumab: 14% (*p* < 0.05 vs. placebo) - Placebo: 4%	NR
GALAXI-1 Danese et al., 2024 [28] Phase 2 RCT maintenance	CD CDAI 220–450 and SES-CD ≥ 3 Inadequate response or intolerance to conventional therapy or biologic therapy	1: Guselkumab SC 100 mg q8w (from IV 200 mg induction) (*n* = 61) 2: Guselkumab SC 200 mg q4w (from IV 600 mg induction) (*n* = 63) 3: Guselkumab SC 200 mg q4w (from IV 1200 mg induction) (*n* = 61) 4: Ustekinumab IV SC 90 mg q8w (*n* = 63)	Clinical remission (CDAI score < 150) at wk 48 - Guselkumab SC 100 mg: 64% - Guselkumab SC 200 mg (from 600 mg IV induction): 73% - Guselkumab SC 200 mg (from 1200 mg IV induction): 57% - Ustekinumab: 59%	Endoscopic response (≥50% improvement in SES-CD or SES-CD ≤ 2) at wk 48 - Guselkumab SC 100 mg: 44% - Guselkumab SC 200 mg (from 600 mg IV induction): 46% - Guselkumab SC 200 mg (from 1200 mg IV induction): 44% - Ustekinumab: 30% Endoscopic remission (SES-CD ≤ 2) at wk 12 - Guselkumab SC 100 mg: 18% - Guselkumab SC 200 mg (from 600 mg IV induction): 17% - Guselkumab SC 200 mg (from 1200 mg IV induction): 33% - Ustekinumab: 6%	NR
GALAXI-2 and -3 Panaccione et al., 2025 [26] Phase 3 RCT maintenance GALAXI-2 and GALAXI-3 had identical study designs	CD CDAI 220–450 and SES-CD ≥ 6, or ≥4 for L1 disease Inadequate response or intolerance to conventional therapy or biologic therapy	1: Guselkumab SC 100 mg q8w (from IV 200 mg induction) (*n* = 286) 2: Guselkumab SC 200 mg q4w (from IV 200 mg induction) (*n* = 296) 3: Ustekinumab IV SC 90 mg q8w (*n* = 291) 4: Placebo (*n* = 148)	Clinical remission (CDAI score < 150) at wk 48 GALAXI-2: - Guselkumab SC 100 mg q8w: 49% (*p* < 0.001 vs. placebo) - Guselkumab SC 200 mg q4w: 55% (*p* < 0.001 vs. placebo) - Placebo: 12% GALAXI-3: - Guselkumab SC 100 mg q8w: 47% (*p* < 0.001 vs. placebo) - Guselkumab SC 200 mg q4w: 48% (*p* < 0.001 vs. placebo) - Placebo: 13%	Endoscopic response (≥50% improvement in SES-CD or SES-CD ≤ 2) at wk 48 GALAXI-2: - Guselkumab SC 100 mg q8w: 27% (*p* < 0.001 vs. placebo) - Guselkumab SC 200 mg q4w: 33% (*p* < 0.001 vs. placebo) - Placebo: 3% GALAXI-3: - Guselkumab SC 100 mg q8w: 24% (*p* < 0.001 vs. placebo) - Guselkumab SC 200 mg q4w: 23% (*p* < 0.001 vs. placebo) - Placebo: 6% Pooled GALAXI-2 and -3 Endoscopic response (≥50% improvement in SES-CD or SES-CD ≤ 2) at wk 48: - Guselkumab SC 100 mg q8w: 48% (*p* < 0.01 vs. ustek) - Guselkumab SC 200 mg q4w: 53% (*p* < 0.001 vs. ustek) - Ustekinumab IV SC 90 mg q8w: 37% Endoscopic remission (SES-CD ≤ 4 and ≥2-point reduction with no single subscore > 1) at wk 48: - Guselkumab SC 100 mg q8w: 33% (*p* = 0.02 vs. ustek) - Guselkumab SC 200 mg q4w: 37% (*p* < 0.01 vs. ustek) - Ustekinumab IV SC 90 mg q8w: 25%	NR
QUASAR Peyrin-Biroulet et al., 2023 [29] Phase 2b RCT induction	UC Adapted mayo score 5–9 points, with rectal bleeding subscore ≥1 and endoscopic subscore ≥ 2 Inadequate response or intolerance to conventional therapy or anti-TNF, VEDO, and/or JAK inhibitor	1: Guselkumab IV 200 mg q4w (*n* = 101) 2: Guselkumab IV 400 mg q4w (*n* = 107) 3: Placebo (*n* = 105)	Clinical remission at wk 12 (a Mayo stool frequency subscore of 0 or 1 and a Mayo rectal bleeding subscore of 0) - Guselkumab 200 mg: 50% (*p* < 0.001 vs. placebo) - Guselkumab 400 mg: 48% (*p* < 0.001 vs. placebo) - Placebo: 20%	Endoscopic response (Mayo endoscopic subscore 0 or 1) at wk 12 - Guselkumab 200 mg: 31% (*p* < 0.05 vs. placebo) - Guselkumab 400 mg: 31% (*p* < 0.001 vs. placebo) - Placebo: 12% Endoscopic remission (Mayo endoscopy subscore of 0) at wk 12 - Guselkumab 200 mg: 18% (*p* < 0.05 vs. placebo) - Guselkumab 400 mg: 14% (*p* = 0.076 vs. placebo) - Placebo: 7%	Histologic improvement (Mayo endoscopic subscore 0 or 1 and geboes score < 3.2) at wk 12 - Guselkumab 200 mg: 20% (p=0.014 vs. placebo) - Guselkumab 400 mg: 27% (*p* < 0.001 vs. placebo) - Placebo: 8.6%
QUASAR Rubin et al., 2025 [30] Phase 3 RCT induction	UC Adapted mayo score 5–9 points, with rectal bleeding subscore ≥1 and endoscopic subscore ≥ 2 Inadequate response or intolerance to conventional therapy or anti-TNF, VEDO, and/or JAK inhibitor	1: Guselkumab IV 200 mg q4w (*n* = 421) 2: Placebo (*n* = 280)	Clinical remission (a Mayo stool frequency subscore of 0 or 1 and a Mayo rectal bleeding subscore of 0) at wk 12 - Guselkumab 200 mg: 23% (*p* < 0.001 vs. placebo) - Placebo: 8%	Endoscopic response (Mayo endoscopic subscore 0 or 1) at wk 12 - Guselkumab 200 mg: 27% (*p* < 0.001 vs. placebo) - Placebo: 11% Endoscopic remission (Mayo endoscopy subscore of 0) at wk 12 - Guselkumab 200 mg: 15% (*p* < 0.001 vs. placebo) - Placebo: 5%	Histologic improvement (Mayo endoscopic subscore 0 or 1 and Geboes score < 3.2) at wk 12 - Guselkumab 200 mg: 45% (*p* < 0.001 vs. placebo) - Placebo: 21% Histological remission (Mayo endoscopic subscore of 0 and Geboes score < 2.1) at wk 12: - Guselkumab 200 mg: 40% (*p* < 0.001 vs. placebo) - Placebo: 19%
QUASAR Rubin et al., 2025 [30] Phase 3 RCT maintenance	UC Participants of QUASAR induction trials who showed clinical response to guselkumab (decrease from induction baseline in Mayo score by at least 30% and at least 2 points)	1: Guselkumab SC 100 mg q8w (*n* = 188) 2: Guselkumab SC 200 mg q4w (*n* = 190) 3: Placebo (*n* = 190)	Clinical remission (a Mayo stool frequency subscore of 0 or 1 and a Mayo rectal bleeding subscore of 0) at wk 44 - Guselkumab 100 mg q8w: 45% (*p* < 0.001 vs. placebo) - Guselkumab 200 mg q4w: 50% (*p* < 0.001 vs. placebo) - Placebo: 19%	Endoscopic response (Mayo endoscopic subscore 0 or 1) at wk 44 - Guselkumab 100 mg q8w: 549% (*p* < 0.001 vs. placebo) - Guselkumab 200 mg q4w: 52% (*p* < 0.001 vs. placebo) - Placebo: 19% Endoscopic remission (Mayo endoscopic subscore = 0) at wk 44 - Guselkumab 200 mg q4w: 35% (*p* < 0.001 vs. placebo) - Guselkumab 100 mg q8w: 34% (*p* < 0.001 vs. placebo) - Placebo: 15%	Histologic improvement (Mayo endoscopic subscore 0 or 1 and Geboes score < 3.2) at wk 44 - Guselkumab 100 mg q8w: 65% (*p* < 0.001 vs. placebo) - Guselkumab 200 mg q4w: 65% (*p* < 0.001 vs. placebo) - Placebo: 31% Histological remission (Mayo endoscopic subscore of 0 and Geboes score < 2.1) at wk 12: - Guselkumab 100 mg q8w: 59% (*p* < 0.001 vs. placebo) - Guselkumab 200 mg q4w: 61% (*p* < 0.001 vs. placebo) - Placebo: 27%
**Mirikizumab (Omvoh)**					
SERENITY Sands et al., 2022 [31] Phase 2 RCT induction	CD Stool frequency ≥4 and/or abdominal pain ≥2 at baseline and a SES-CD ≥ 6 and ≥4 with L1 disease Inadequate response or intolerance to conventional therapy or biologic therapy	1: Mirikizumab IV 200 mg q4w (*n* = 31) 2: Mirikizumab IV 600 mg q4w (*n* = 32) 3: Mirikizumab IV 1000 mg q4w (*n* = 64) 4: Placebo (*n* = 64)	Clinical remission (CDAI score < 150) at wk 12: - Mirikizumab 200 mg: 16% (*p* = 0.41 vs. placebo) - Mirikizumab 600 mg: 41% (*p* < 0.001 vs. placebo) - Mirikizumab 1000 mg: 27% (*p* = 0.032 vs. placebo) - Placebo: 9%	Endoscopic response (SES-CD 50% reduction) at wk 12: - Mirikizumab 200 mg: 26% (*p* = 0.079 vs. placebo) - Mirikizumab 600 mg: 38% (*p* = 0.003 vs. placebo) - Mirikizumab 1000 mg: 44% (*p* < 0.001 vs. placebo) - Placebo: 11% Endoscopic remission (SES-CD < 4 for ileocolonic disease or <2 for L1 disease, no subscore > 1) at wk 12: - Mirikizumab 200 mg: 7% (*p* = 0.241 vs. placebo) - Mirikizumab 600 mg: 16% (*p* = 0.032 vs. placebo) - Mirikizumab 1000 mg: 20% (*p* = 0.009 vs. placebo) - Placebo: 2%	NR
SERENITY Sands et al., 2022 [31] Phase 2 RCT maintenance	CD Participants of the SERENITY induction trials who achieved an improvement of at least 1 point decrease in their SES-CD score	1: Mirikizumab IV dose continued as received during induction q4w(*n* = 41) 2: Mirikizumab SC 300 mg q4w (*n* = 46)	Clinical remission (CDAI score < 150) at wk 52: - Mirikizumab IV dose as received during induction: 39% - Mirikizumab SC 300 mg: 57%	Endoscopic response (SES-CD 50% reduction) at wk 52 - Mirikizumab IV dose as received during induction: 59% - Mirikizumab SC 300 mg: 59% Endoscopic remission (SES-CD < 4 for ileocolonic disease or <2 for L1 disease, no subscore > 1) at wk 52 - Mirikizumab IV dose as received during induction: 20% - Mirikizumab SC 300 mg: 32%	NR
VIVID-1 Ferrante et al., 2024 [32] Phase 3 RCT maintenance	CD Stool frequency ≥ 4 and/or abdominal pain ≥2 at baseline and a SES-CD ≥6 and ≥4 with L1 disease Inadequate response or intolerance to conventional therapy or biologic therapy	1: Mirikizumab IV 900 mg w4–8, then 300 mg SC q4w (*n* = 579) 2: ustekinumab 6 mg/kg IV at w0, then 90 mg SC q8w (*n* = 287) 3: Placebo (*n* = 199)	Clinical remission (CDAI score < 150) at wk 52: - Mirikizumab 900 mg: 45% (*p* < 0.001 vs. placebo) - Placebo: 20%	Endoscopic response (SES-CD 50% reduction) at wk 52 - Mirikizumab 900 mg: 38% (*p* < 0.001 vs. placebo) - Placebo: 9% Endoscopic remission (SES-CD <4 for ileocolonic disease or <2 for L1 disease, no subscore > 1) at wk 52 - Mirikizumab 900 mg: 16% (*p* < 0.001 vs. placebo) - Placebo: 2%	NR
Sandborn et al., 2020 [33] Phase 2 RCT induction	UC Mayo score of 6–12, including an endoscopic subscore ≥ 2 No previous exposure to therapy targeting IL-23 pathway	1: Mirikizumab IV 50 mg q4w (*n* = 63) 2: Mirikizumab IV 200 mg q4w (*n* = 62) 3: Mirikizumab IV 600 mg q4w (*n* = 61) 4: Placebo (*n* = 63)	Clinical remission (Mayo subscore = 0 for rectal bleeding, 0 or 1 for stool frequency) at wk 12: - Mirikizumab 50 mg: 15% (*p* = 0.066 vs. placebo) - Mirikizumab 200 mg: 23% (*p* = 0.004 vs. placebo) - Mirikizumab 600 mg: 12% (*p* = 0.14 vs. placebo) - Placebo: 5%	Endoscopic response (Mayo endoscopic subscore of 0 or 1) at wk 12 - Mirikizumab 50 mg: 24% (*p* = 0.012 vs. placebo) - Mirikizumab 200 mg: 31% (*p* < 0.001 vs. placebo) - Mirikizumab 600 mg: 13% (*p* = 0.22 vs. placebo) - Placebo: 6% Endoscopic remission (Mayo endoscopic subscore = 0) at wk 12: - Mirikizumab 50 mg: 3% (*p* = 0.56 vs. placebo) - Mirikizumab 200 mg: 3% (*p* = 0.55 vs. placebo) - Mirikizumab 600 mg: 2% (*p* = 0.99 vs. placebo) - Placebo: 2%	Histological remission (Geboes histologic subscores of 0 for the neutrophils in lamina propria, neutrophils in epithelium, and erosion or ulceration parameters) at wk 12: - Mirikizumab 50 mg: 14% (*p* = 0.632 vs. placebo) - Mirikizumab 200 mg: 45% (*p* = 0.001 vs. placebo) - Mirikizumab 600 mg: 34% (*p* = 0.028 vs. placebo) - Placebo: 14%
LUCENT-1 D’Haens et al., 2023 [34] Phase 3 RCT induction	UC Adapted mayo score 4–9 points and endoscopic subscore ≥ 2 Inadequate response or intolerance to conventional therapy or biological therapy	1: Mirikizumab IV 300 mg q4w (*n*= 868) 2: Placebo (*n*= 294)	Clinical remission (≥1-point decrease in stool frequency subscore or subscore 0 or 1, and rectal bleeding subscore of 0) at wk 12 - Mirikizumab 300 mg: 24% (*p* < 0.001 vs. placebo) - Placebo: 13%	Endoscopic remission (Mayo endoscopic subscore 0 or 1) at wk 12 - Mirikizumab 300 mg: 36% (*p* < 0.001 vs. placebo) - Placebo: 21%	Histologic improvement (Mayo endoscopic subscore 0 or 1 + neutrophil infiltration in crypts <5% and absence of crypt destruction, ulcerations, erosions, and granulation tissue) at wk 12 - Mirikizumab 300 mg: 27% (*p* < 0.001 vs. placebo) - Placebo: 14%
LUCENT-2 D’Haens et al., 2023 [34] Phase 3 RCTmaintenance	UC All the patients who completed the induction trial, regardless of clinical response, were eligible to participate in the maintenance trial	1: Mirikizumab SC 200 mg (*n*= 365) 2: Placebo (*n*= 179)	Clinical remission (≥1-point decrease in stool frequency subscore or subscore 0 or 1, and rectal bleeding subscore of 0) at wk 40: - Mirikizumab 200 mg: 50% (*p* < 0.001 vs. placebo) - Placebo: 25%	Endoscopic remission (Mayo endoscopic subscore 0 or 1) at wk 40: - Mirikizumab 200 mg: 59% (*p* < 0.001 vs. placebo) - Placebo: 29%	Histologic improvement (Mayo endoscopic subscore 0 or 1 + neutrophil infiltration in crypts <5% and absence of crypt destruction, ulcerations, erosions, and granulation tissue) at wk 40 - Mirikizumab 200 mg: 49% (*p* < 0.001 vs. placebo) - Placebo: 25%
LUCENT-3 Sands et al., 2024 [35] Phase 3 maintenance open-label extension trial	UC	1. Mirikizumab SC 200 mg q4w	Clinical remission at wk 104 (based on modified Mayo score): wk 52 responders: 54.0% wk 52 remitters: 65.6%	Endoscopic remission (Mayo endoscopic subscore 0 or 1) at wk 104: wk 52 responders: 65.3% wk 52 remitters: 77.3% Endoscopic/histologic remission at wk 104: wk 52 responders: 47.7% wk 52 remitters: 74.0%	
